# A respirometry system designed for small ruminants

**DOI:** 10.3168/jdsc.2025-0755

**Published:** 2025-07-17

**Authors:** C.S. Silva, J. Diavão, E.F. Motta, A.S. Silva, R.G. Tonucci, T.R. Tomich, F.S. Machado, M.M. Campos

**Affiliations:** Brazilian Agricultural Research Corporation, Juiz de Fora, Minas Gerais, Brazil, 36038-330

## Abstract

This technical note describes a small ruminant respiration chamber system designed to accurately quantify the production of carbon dioxide (CO_2_) and methane (CH_4_). The system consists of 3 open-circuit respiration chambers, flow meters, gas analyzers, and an accessible environmental control system. To validate its performance, gas recovery tests were conducted by injecting CO_2_ and CH_4_ at 4 constant flow rates: 0.4, 0.8, 1.2, and 1.6 standard liters per minute (SLPM) for CO_2_, and 0.16, 0.20, 0.24, and 0.28 SLPM for CH_4_. Each injection level was tested across the 3 chambers. The average percentage recoveries for CO_2_ and CH_4_ were 97.8% ± 1.46% for chamber 1, 101.1% ± 1.49% for chamber 2, and 104.1% ± 1.98% for chamber 3 and 98.7% ± 0.97% for chamber 1, 99.4% ± 2.06% for chamber 2, and 101.6% ± 2.90% for chamber 3, respectively, demonstrating the system's accuracy in quantifying livestock GHG emissions from small ruminants, such as young cattle, goats, and sheep. Therefore, this system represents a viable methodology for nutritional and environmental studies with small ruminants. Future studies conducted in this facility can contribute to a better understanding of the dynamic and flow of GHG emissions in small ruminants and target measures to increase the sustainability of small ruminant production systems.

## Highlights

• Respiration chambers can be costly and require extensive maintenance.
• We describe a simpler facility for respirometry studies with small ruminants.
• The system was validated through gas recovery tests, demonstrating reliable performance.
• The system can effectively measure gas emissions from small ruminant species.

## Graphical Abstract

**Figure:**
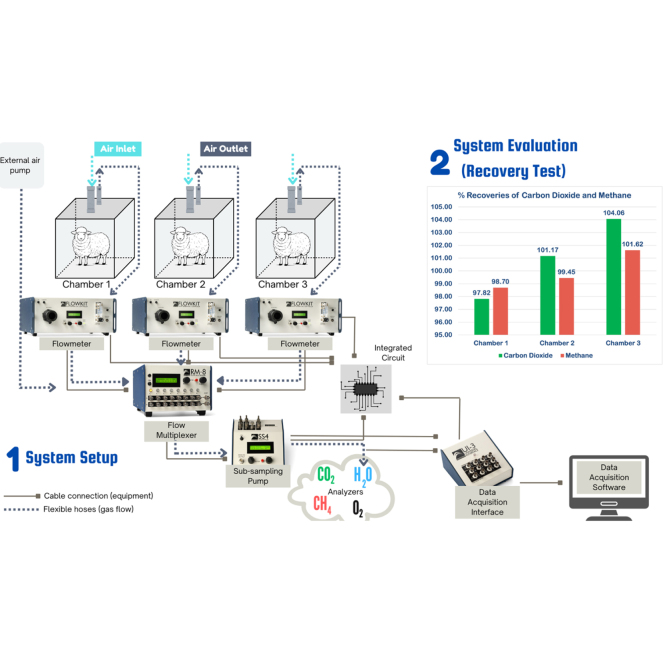
**Summary:** The respirometry system presented herein consists of 3 open-circuit respiration chambers, a set of flowmeters, and gas analyzers for water vapor (H_2_O), carbon dioxide (CO_2_), methane (CH_4_), and oxygen (O_2_). The chambers are connected to air inlet and outlet pipes, delivering ambient and chamber air, respectively. Chamber air is continuously extracted and sampled by the flowmeters. Both chamber and external air samples are automatically directed to the flow multiplexer, then subsampled by a pump and analyzed for H_2_O, CO_2_, CH_4_, and O_2_ concentrations. The analyzed values, along with barometric pressure and other measurement conditions, are recorded using a data acquisition software, and can be exported for gas exchange calculations. To validate the system, gas recovery tests were conducted by injecting CO_2_ and CH_4_ at different levels and constant flow rates. The average recovery percentages ranged from 97.82% to 104.06% for CO2 and 98.70% to 101.62% for CH_4_ across all 3 chambers.

Livestock is a major contributor to GHG emissions, accounting for 7.1 billion tonnes of carbon dioxide (CO_2_) equivalent annually, or 14.5% of all human-induced emissions (FAO, 2024). Enteric fermentation, particularly methane (CH_4_) production, is the largest source of these emissions; more importantly, CH_4_ has a global warming potential 28 to 36 times greater than that of CO_2_ over a 100-year period (IPCC, 2023). Therefore, developing nutritional strategies that enhance feed efficiency in domestic ruminants while reducing CH_4_ output is key. This requires precise measurements of gaseous exchanges from fermentation and nutrient oxidation, highlighting the importance of specialized tools such as open-circuit respiration chambers.

Respiration chambers are widely recognized as the “gold standard” for assessing gaseous exchanges and energy metabolism in ruminants (Troy et al., 2016). Although predominantly used in studies with large ruminants, accounting for 56% of applications (Della Rosa et al., 2021), respiration systems designed for small ruminants offer substantial potential. Their advantages include lower experimental costs and labor requirements, the use of small ruminants as models to refine energy feed fraction analysis—thereby reducing errors in diet formulation—and the selection of efficient animals through precise measurements of dietary energy losses, heat production, and energy retention (VandeHaar, 2016). Small respiration systems help validate conventional models that may overlook specific animal types, causing estimation errors. They also enable the assessment of CH_4_ and CO_2_ emissions and support research aimed at reducing the environmental impact of small ruminant production.

This technical note introduces a cost-effective and simplified respiration system specifically designed for studies involving small ruminants. We hypothesized that this system can successfully recover CO_2_ and CH_4_ emitted by small ruminants, such as young cattle (up to 200 kg), goats, and sheep. Our objective is to describe and validate this system through recovery tests of CH_4_ and CO_2_, offering an accessible and reliable alternative for measuring small ruminant GHG emissions.

The respiration chambers are housed in the Multiuser Laboratory for Livestock Bioefficiency and Sustainability at the Brazilian Agricultural Research Corporation, Embrapa, located in Coronel Pacheco, Minas Gerais, Brazil (https://www.embrapa.br/laboratorio-multiusuario-de-bioeficiencia-e-sustentabilidade-da-pecuaria). The system is set up as an open-circuit respiration system consisting of 3 chambers (Ponta, Betim, MG, Brazil), a set of flow meters (FlowKit FK-500, Sable Systems International, Las Vegas, NV), and CO_2_ (CA-10 Carbon Dioxide, Sable Systems International), CH_4_ (MA-10 CH_4,_ Sable Systems International), and water vapor (RH-300, Sable Systems International) analyzers. The MA-10 CH_4_ and CA-10 CO_2_ models employ dual-wavelength nondispersive infrared detection, utilizing a solid-state, electrically modulated true blackbody infrared source. The chambers' walls are made of aluminum and transparent polyethylene terephthalate glycol to allow visual contact between animals (Figure 1), minimizing animal stress while enhancing internal visualization for the chamber operator.

**Figure 1:**
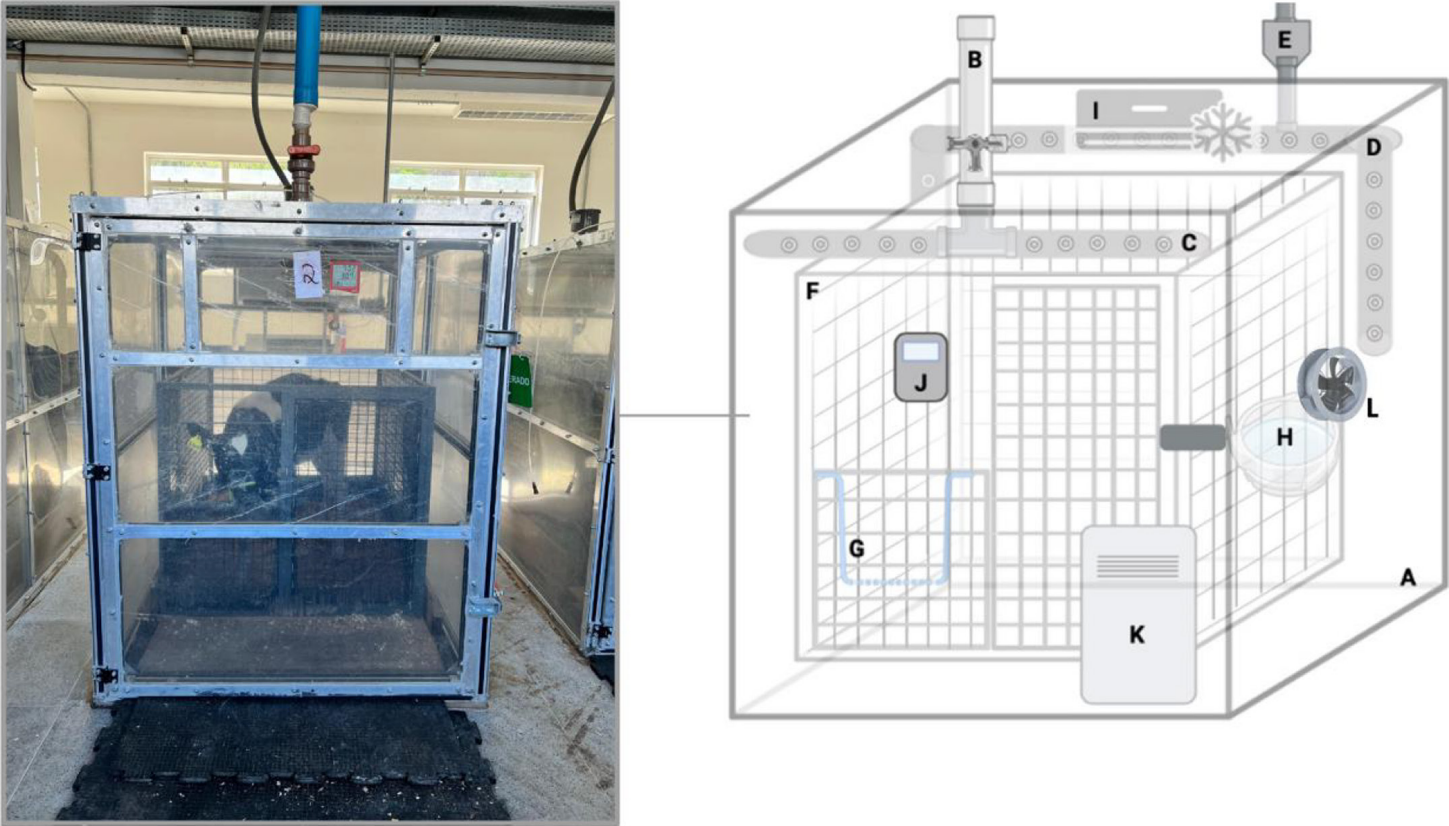
Diagram of the respirometry chamber designed for small ruminants. A: respirometry chamber (aluminum-polyethylene structure); B: air inlet pipe; C: punctured PVC pipes connected to air inlet; D: punctured PVC pipes connected to air outlet; E: air outlet and filter box; F: metal cage; G: feed bin attached to metal cage; H: water trough attached to metal cage; I: air conditioning unit; J: thermo-hygrometer; K: dehumidifier; L: air circulation fan attached to internal chamber wall. Created in BioRender.com.

The chambers are fitted with one rubber-sealed front door for animal access. Fresh air is drawn from outside the building into the chamber through a 75-mm-diameter polyvinyl chloride (**PVC**) pipe connected to fresh air inlets onto the front end of the ceiling (Figure 1B). Inside the chamber, the fresh air inlet is fitted with a valve and a T-connection connected by 2 horizontal PVC tubes (50 mm diameter × 1.34 m) punctured with 1-cm holes to avoid laminar flow (Figure 1C). The chamber's air is continuously collected into 75-mm PVC pipes, also punctured with 1-cm holes (Figure 1D), connected to an air outlet and a filter box (CSL-849–100HC, Solberg Manufacturing Inc., Itasca, IL), placed on the rear section of the ceiling (Figure 1E). These are directly coupled to a mass flow meter integrated with a sealed rotary pump capable of sustaining an airflow rate from 75 to 500 L/min (FlowKit Model FK-500, Sable International Systems, Las Vegas, NV).

The chambers are provided with a metal cage (1.85 m long × 1.14 m wide × 1.04 m high; Ponta, Betim, MG, Brazil) where animals are placed during respiration measurements (Figure 1F). The cage is enclosed with a galvanized steel screen, has a rubber mat floor, and is equipped with a feed bin and drinker (Figure 1G and H). An air-conditioning unit is installed in the upper-middle section of the chamber (Figure 1I) to regulate the internal temperature, which is continuously monitored using a digital thermo-hygrometer (Figure 1J; THU-100, Unity Instrumentos, São Paulo, SP, Brazil). A dehumidifier (model 160, Arsec Desumidificadores, Vargem Grande Paulista, SP, Brazil), placed next to the front door inside the chamber (Figure 1K), is used to control humidity. To ensure uniformity, the chamber air is constantly circulated by a fan (Figure 1L). The net volume of the chambers is 5.95 m^3^ (2.41 m long × 1.42 m wide × 1.74 m high). The internal pressure of the chambers is measured each morning before the commencement of gas flow measurements using a digital pressure indicator (DPI 705, Druck Limited, Leicester, UK). This procedure is conducted to verify the integrity of the chamber seal and ensure there is no gas leakage. A slight negative pressure reading on the indicator confirms that the chamber is adequately sealed. After streaming up the air outlet, the air flows through a 51-mm-diameter flexible polyurethane hose, next to the analysis room, and is collected directly by the mass flow meter. The flow is typically set at 1 L/kg of BW per min but can be adjusted based on the specific conditions of the animals undergoing respirometry evaluations (e.g., maintenance vs. production levels). Most importantly, the selected flow rate must ensure accurate gas concentration measurements by the analyzers, safeguard the animals from elevated CO_2_ levels, maintain measurable concentrations of target gases, and generate negative pressure within the chambers to prevent gas leakage. Accordingly, all chambers operate under negative pressure.

The flow meter, which measures incoming air from the chambers, is set to a flow rate of 0.5 L/min, with acceptable variations ranging from 0.45 to 0.55 L/min. This subsampled flow is directed to an 8-channel gas switching system (RM-8 Flow Multiplexer, Sable Systems International). There, a subsampling pump draws gas at 0.2 L/min through a sampling manifold (T-connection). Ambient (baseline) air is also continuously sampled at the same flow rate by a positive pressure pump (B-pump, Sable Systems International).

The multiplexer automatically switches from one chamber air stream to another or to external air during recordings at a defined interval, which is set via the data acquisition software Expedata (Expedata Data Analysis Software 1.9.27, version PRO, Sable Systems International). The system is currently programmed to switch channels every 300 s, from baseline (external air) to chambers 1, 2, and 3 and back to baseline (Figure 2). Therefore, the time required for a full measuring cycle with the 3 chambers working simultaneously is 20 min.

**Figure 2:**
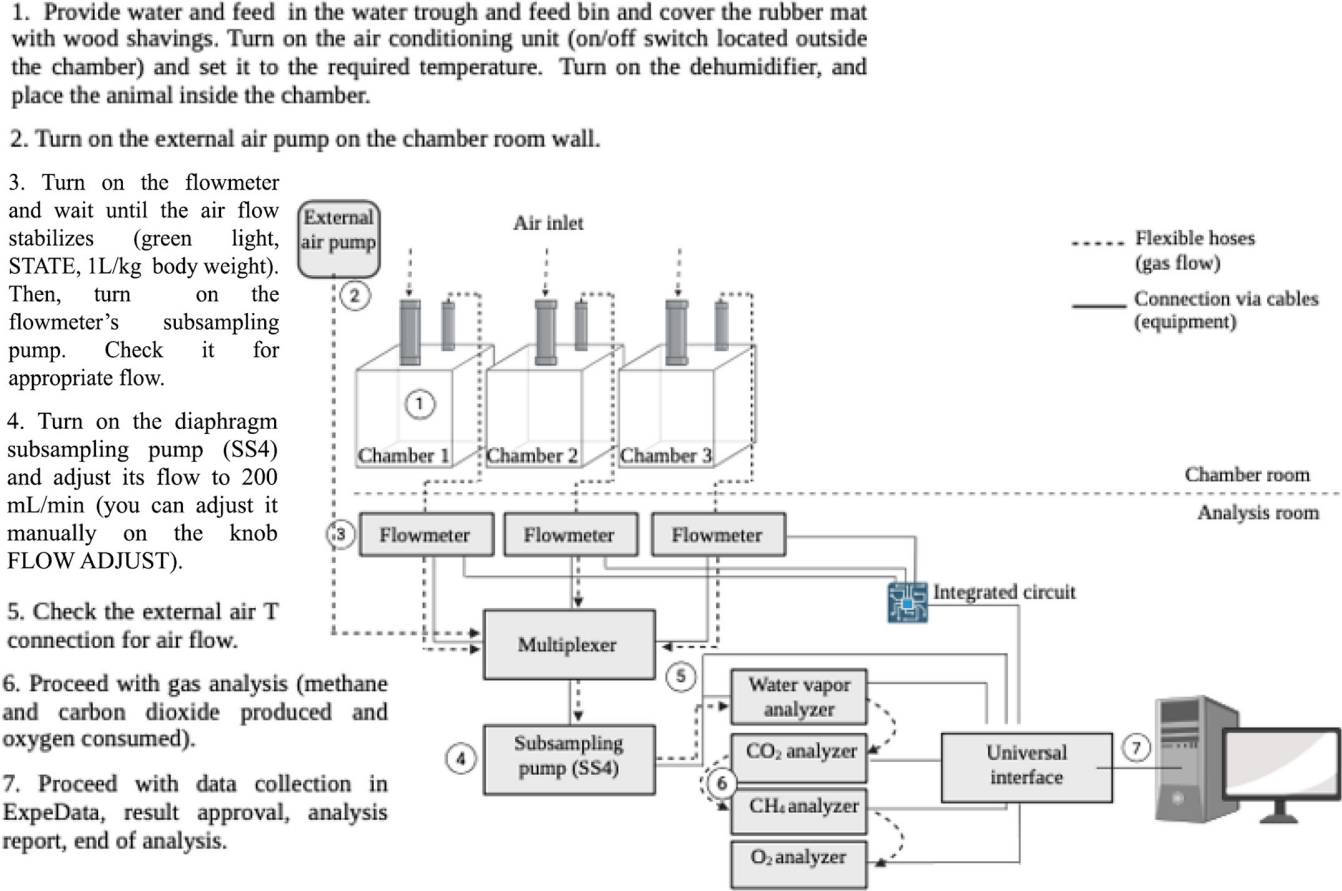
Schematic diagram of the respirometry system designed for small ruminants and critical steps for proper gas exchange measurements. Created in BioRender.com.

Chamber and baseline air samples are individually delivered by the multiplexer to a diaphragm subsampling pump (SS4 Sub-Sampler Pump, Sable Systems International), which sequentially delivers the respective subsample to water vapor (RH-300 Water Vapor Analyzer, Sable Systems International), CO_2_, and CH_4_ analyzers at a flow rate of 0.2 L/min. All tubing from the subsampling pumps to the gas analyzers uses Bev A-Line IV tubes, size B5 (Excelon, Long Hill Township, NJ). The gas analyzers have internal temperature control and barometric pressure compensation, so the readout is already corrected to standard temperature and pressure (**STP**) conditions. The detection range, absolute accuracy, and resolution for relative humidity, CO_2_, and CH_4_ are humidity (detection range: 0%–100% range, accuracy: 2%, resolution: 0.001%), CO_2_ (0–10%, accuracy: 1%, resolution: 0.0001%), and CH_4_ (0%–10%, accuracy: 1%, resolution: 0.001%).

The gas analyzers must be calibrated (zeroed and spanned) both before and during respiration trials, following the instructions provided in each device's user manual. The CO_2_ and CH_4_ analyzers are calibrated daily, before the start of measurements, whereas the water vapor analyzer is calibrated once a week. The weekly calibration schedule for the water vapor analyzer accounts for environmental variations, such as changes in temperature and humidity, which can fluctuate between weeks and influence the characteristics of the sampled air (Lighton, 2018). The CO_2_ and CH_4_ analyzers are zeroed using nitrogen gas with 99.999% purity, and spanned with a calibration gas mixture containing 0.5% CO_2_ and 0.1% CH_4_ in nitrogen as the carrier gas. The water vapor analyzer is zeroed with the dry ambient air scrubbed of moisture with magnesium perchlorate [Mg(ClO_4_)_2_; Smith, 1962], and the span value is calculated using the equation proposed by Lighton (2018):

WVP = BP × [(F'iO_2_ − FiO_2_)/FiO_2_],

where WVP is the water vapor pressure in the same units as barometric pressure (kPa); BP is the barometric pressure; and F'iO_2_ and FiO_2_ are O_2_ fractional concentrations of dry and wet ambient air, respectively.

Measurement conditions such as wet air flow rate, temperature, barometric pressure, water vapor pressure, and gas concentrations are recorded by the software Expedata via a data acquisition interface. The software uses a macro utility to correct the effect of water vapor, lag time, and drift on flow rate and gas concentrations (Machado et al., 2016). The recorded data are exported to an Excel (Microsoft Corp.) spreadsheet and used to calculate CO_2_ and CH_4_ production. To ensure reliable results, animals are assessed over 24 h in each chamber by 2 consecutive days, and the average CO_2_ and CH_4_ values are used as the final respiration measurements. Feed intake within the chamber is compared with the animal's average intake during the week preceding the respiration trial. If intake drops by more than 5%, the respiration measurements are repeated until 2 validated days are obtained, as described by Machado et al. (2016).

A recovery test is performed on the entire system before and after data acquisition as recommended by Mesgaran et al. (2020). The test involves injecting known volumes of CO_2_ (99.990% purity, White Martins, Rio de Janeiro, RJ, Brazil) and CH_4_ (99.995% purity, White Martins) separately into each chamber for 4 h and calculating the volume of gas recovered after 22 h. This was selected to reflect the experimental routine, as the gas exchange measurements accommodate essential management tasks—such as changing the bedding, cleaning feeders and drinkers, assessing animal health, feeding, and sample collection if necessary—that occur daily from 0600 to 0800 h.

In routine gas recovery checks, the flow rates for gas injections are typically set at 1.0 L/min for CO_2_ and 0.25 mL/min for CH_4_. In our laboratory, the flow rate is regulated by a mass flow meter with a maximum flow rate of 50 L/min (MC-50SLMP-D/5M, Alicat Scientific Inc., Tucson, AZ). For the present validation, we conducted a series of recovery tests using 4 levels of injection CO_2_ (0.4, 0.8, 1.2, and 1.6 standard liters per minute; **SLPM**) and CH_4_ (0.16, 0.20, 0.24, and 0.28 SLPM) across the 3 chambers. These injection volumes were selected to represent the average daily CO_2_ and CH_4_ emissions from suckling calves (Lage et al., 2019), weaned calves (L. D. Ferreira, Federal University of Minas Gerais, Belo Horizonte, Brazil; unpublished data), and adult sheep (Sousa et al., 2022), based on previous experiments carried out at the same facility using these 3 animal categories.

The recovery tests were conducted following the standard operating procedure, after calibrating the water vapor and gas analyzers. Expedata files generated after 22 h of reading were processed using the macro utility feature of the software. Upon completion of the analysis, the software generated an Excel spreadsheet containing the percentages of CO_2_ and CH_4_ in outgoing (chamber air) and incoming (ambient) air samples, as well as the flow rate, set as 100 L/min, for each cycle (5 min) with a temperature inside the chamber set as 22°C.

These concentrations were then used to calculate the chambers' correction factor for CO_2_ or CH_4_ production (i.e., injection) using the following formula:

gasproductionCO2orCH4,LLminmin=gasvolumeoutgoingair×outgoingSTPD-gasvolumeingoingair×ingoingSTPD,

where gas volume in outgoing and ingoing air correspond to ingoing CO_2_ (CO_2_i%), ingoing CH_4_ (CH_4_i%), and outgoing CO_2_ (CO_2_e%) or outgoing CH_4_ (CH_4_e%) in the Expedata report sheet, divided by 100; outgoing standard temperature pressure and dry volume (**SPTD**) represents the flow rate (L/min); and ingoing SPTD corresponds to the outgoing SPTD × nitrogen factor. The SPTD conditions were estimated based on chamber volume, pressure of 101.325 kPa, and 273 kelvin.

When the volumes of O_2_ consumed and CO_2_ produced are not equal (e.g., the respiratory quotient is not 1.00) or gasses like CH_4_ are emitted to chamber air (or both), the ingoing and outgoing air volume will differ. To correct for this, the ingoing airflow is calculated as the outgoing standard temperature and pressure dry (**STPD**) multiplied with the nitrogen factor. The nitrogen factor is the ratio between the concentration of nitrogen gas leaving and entering the respiration chamber. This calculation assumes that the same amount of STPD N_2_ gas enters and leaves the chamber. The concentrations of nitrogen gas are calculated as 100% minus the concentrations of O_2_, CO_2_, and CH_4_ (Mesgaran et al., 2020).

The nitrogen (N) factor was calculated as

$$\begin{array}{c} N\;\mathrm{factor}\;\left(\&,p,e,r,c,n,t,;\right)=\\ \;\frac{\left(100-\;CO_{2}\mathrm{outgoing}-\;O_{2}\mathrm{outgoing}-\;CH_{4}\mathrm{outgoing}\right)}{\left(100-\;CO_{2}\mathrm{ingoing}-\;O_{2}\mathrm{ingoing}-\;CH_{4}\mathrm{ingoing}\right)}. \end{array}$$

The gas production calculated in the previous equation was used to determine the gas production per cycle (L/cycle), where

$$\begin{array}{c} \mathrm{gas}\;\mathrm{production}\;\mathrm{per}\;\mathrm{cycle}\;\left(L/\mathrm{cycle}\right)=\\ \mathrm{gas}\;\mathrm{production}\;\left(L/\min\right)\times \mathrm{time}\;\mathrm{per}\;\mathrm{cycle}\;\left(\min\right). \end{array}$$

Considering that each cycle corresponds to a 10-min run for one chamber (i.e., chamber air plus baseline), the time per cycle used in the above equation was 10, 15, or 20 min, depending on the number of chambers being evaluated simultaneously. The gas production per cycle was then used to estimate the 22-h cumulative gas production (i.e., injection) as follows:

$$P_{\mathrm{cumulative}\left(n\right)}=\;P_{n}+P_{\mathrm{cumulative}\left(n-1\right)},$$

where P_cumulative(_*_n_*_)_ is the cumulative gas production at cycle *n*, P*_n_* is the gas production in cycle *n*, P_cumulative(_*_n_*_−1)_ is the cumulative gas production up to the previous cycle (n − 1).

To start the calculation, the value of the first cycle (P_cumulative(1)_) was equal to the production of the cycle itself:

$$P_{\mathrm{cumulative}\left(1\right)}=P1.$$

After the cumulative gas production was determined, the volume of gas recovered from an individual chamber (*i*) was given as the maximum value of cumulative gas production in the respective chamber. Last, the gas recovery percentage (correction factor) was calculated as

$$\begin{array}{c} \mathrm{correction}\;\mathrm{factor}\;\left(\&,p,e,r,c,n,t,;\right)=\\ \left(\frac{\mathrm{volume}\;\mathrm{of}\;\mathrm{gas}\;\mathrm{recovered}\;\mathrm{from}\;\mathrm{chamber}\;i}{\mathrm{volume}\;\mathrm{of}\;\mathrm{gas}\;\mathrm{injected}\;\mathrm{into}\;\mathrm{chamber}\;i}\right)\times 100. \end{array}$$

Gerrits et al. (2018) emphasized the importance of conducting and reporting recovery tests for respiration trial results to ensure acceptable recovery ranges, whether using chamber or nonchamber techniques. Gardiner et al. (2015), for instance, used an ultra-high-purity CH_4_ standard and examined 3 sources of experimental error—analyzer error, ducting efficiency (including airflow measurements), and chamber mixing—to evaluate the gas recoveries in 22 chambers across 6 facilities. There was a large variation in gas recovery for the different chamber systems and testing conditions, with recovery percentages ranging from 59% to 115%.

In our current recovery tests, average recoveries across different CO_2_ and CH_4_ injection levels were 98.26% ± 0.87% for chamber 1, 100.31% ± 1.59% for chamber 2, and 102.84% ± 1.79% for chamber 3 (Table 1). Unlike Gardiner et al. (2015), our results show minimal deviation from complete gas recovery, indicating that our chamber system provides accurate measurements of gas emissions from ruminants weighing up to 200 kg, which typically emit an average 88–429 L/d of CO_2_ and 34–64 L/d of CH_4_. However, due to the limitations of our mass flow controller, we were unable to quantify lower CH_4_ injection volumes (<0.16 SPLM). Therefore, we cannot confirm reliability for CH_4_ emissions below 33 L/d.

**Table 1:** Gas recovery for carbon dioxide (CO_2_) and methane (CH_4_) injected into a respiration system with 3 small chambers

Chamber no. and selected gas	Flow rate (SLPM^1^)	Injected gas volume (L)	Recovered gas volume (L)	Recovery (%)
1				
CO_2_	0.40	94.76	90.66	95.67
CO_2_	0.80	175.78	173.4	98.65
CO_2_	1.20	291.43	285.98	98.13
CO_2_	1.60	429.17	424.19	98.84
CH_4_	0.16	35.93	35.09	97.66
CH_4_	0.20	44.25	43.4	98.08
CH_4_	0.24	57.3	57.1	99.65
CH_4_	0.28	62.12	61.75	99.40
2				
CO_2_	0.40	96.6	95.8	99.17
CO_2_	0.80	180.48	183.99	101.94
CO_2_	1.20	267.39	274.36	102.61
CO_2_	1.60	335.8	338.95	100.94
CH_4_	0.16	35.45	35.05	98.87
CH_4_	0.20	43.65	42.4	97.14
CH_4_	0.24	54.66	54.49	99.69
CH_4_	0.28	62.96	64.29	102.11
3				
CO_2_	0.40	88.4	89.96	101.76
CO_2_	0.80	179.37	185.92	103.65
CO_2_	1.20	266.69	278.08	104.27
CO_2_	1.60	352.54	375.70	106.57
CH_4_	0.16	34.86	33.95	97.39
CH_4_	0.20	44.02	45.12	102.50
CH_4_	0.24	52.85	54.21	102.57
CH_4_	0.28	61.76	64.25	104.03

1 Standard liters per minute.

The highest CO_2_ recovery observed (106.57% at a 1.60 SPLM flow rate) was due to an inward CO_2_ leak from the chamber floor, as later confirmed scrubbing the floor with soap. Such gas contamination can lead to overestimations in open-circuit respiration chambers (Gardiner et al., 2015). Recovery tests should ideally be repeated whenever CO_2_ or CH_4_ recoveries fall below 95% or exceed 105%.

During system validation, several critical control points and potential sources of error were identified that should be routinely monitored in similar chamber-based experimental systems. These include the following: (1) Inspect door seals, pipes, wall structure, and bolts for leaks. (2) Check the chamber floor for cracks or porosity that could lead to gas leaks. (3) Ensure the mass flow meter meets injection requirements; the injection level may be lower than the equipment's minimum flow control. (4) Calibrate analyzers correctly, especially zeroing and spanning the water vapor analyzer to prevent incorrect readings. (5) Confirm that flow readings match the set point on the chamber's flow meter (FlowKit). (6) Verify calculations to ensure all input parameters align with test settings (e.g., number of chambers analyzed, reading time for a full cycle, chamber transitions). (7) Adjust input parameters in Expedata and macros to match current test conditions.

Our results demonstrate that the described respiration system is a reliable and effective tool for quantifying greenhouse gas emissions in small ruminant research, including studies focused on emission profiling and evaluating mitigation strategies.
